# Peer Worker-Supported Transition from Hospital to Home—Outcomes for Service Users

**DOI:** 10.3390/ijerph19052743

**Published:** 2022-02-26

**Authors:** Nicola Hancock, Bridget Berry, Michelle Banfield, Georgia Pike-Rowney, Justin Newton Scanlan, Sarah Norris

**Affiliations:** 1 Faculty of Medicine and Health, The University of Sydney, Camperdown 2006, Australia; bridget.berry@sydney.edu.au (B.B.); justin.scanlan@sydney.edu.au (J.N.S.); sarah.norris@sydney.edu.au (S.N.); 2 Centre for Disability Research and Policy (CDRP), The University of Sydney, Camperdown 2006, Australia; 3 College of Health and Medicine, Australian National University, Canberra 0200, Australia; michelle.banfield@anu.edu.au (M.B.); georgia.pike@anu.edu.au (G.P.-R.)

**Keywords:** co-production, peer support, recovery, discharge, hospital avoidance, lived experience research

## Abstract

Background: Transitioning from psychiatric hospitalisation back to community presents a period of heightened suicide, homelessness, relapse, and rehospitalisation risk. The Australian state of New South Wales established a state-wide Peer Supported Transfer of Care (Peer-STOC) initiative to enhance recovery-focused supports available during this transition period. Aims: To understand the impacts and outcomes of the Peer-STOC program on service users from three stakeholder perspectives: service users themselves, peer worker service providers, and other mental health workers and clinicians interfacing with the program. Methods: Qualitative data from 82 questionnaires and 58 individual in-depth interviews were analysed thematically using constant comparative methods and an iterative and inductive process. Results: All stakeholders described positive impacts and outcomes of the program for service users. These included: (a) a better, less traumatic inpatient experience; (b) felt understood, cared about and less alone; (c) easier to leave hospital; (d) easier to get back into life and daily routines; (e) built and re-established community connections; (f) gained new knowledge, strategies, and skills; and (g) felt more hopeful about my recovery. Conclusions: The Peer-STOC program had a positive impact. It enhanced people’s experience in hospital, eased their transition from hospital and assisted with people recovering community-based relationships, activities, and routines.

## 1. Introduction

Emerging evidence from increasingly rigorous studies demonstrate that a peer workforce can positively impact on service-user outcomes [1,2,3,4,5]. Davidson and colleagues’ [6] synthesis of evidence found positive effects of peer support across numerous domains including: engaging people in caring relationships; improving relationships between service-users and outpatient providers; increasing engagement in non-acute, less costly care; decreasing substance use; decreasing unmet needs; and increasing recovery and quality of life.

Peer Supported Transfer of Care (Peer-STOC) is an Australian, state-wide initiative funded by the New South Wales Ministry of Health. Peer-STOC is designed to provide peer worker-led, recovery-focused support to people with complex mental health needs during their transition to home or community after an inpatient admission.

Primary drivers that led to Peer-STOC related to identified problems needing resolution and identified opportunities and emerging evidence. Poor outcomes for people transitioning to community from acute adult mental health in-patient care was one primary driver. Poor outcomes were evidenced first by the high rates of re-admission soon after discharge [7]. Almost 15% of people were rehospitalised within 28 days of discharge from psychiatric hospitalisation during 2019–20 [8]. Second, there is a heightened risks of homelessness and suicide in the early period post-discharge from hospital to community, particularly for those living with complex mental illness and those not engaged with health care services [9,10].

In addition to these heightened risks for people being discharged, community mental health teams were struggling to follow up post-discharge. A lack of connection with community-based support is a significant factor associated with the poor outcomes described above [11,12].

Alongside the above need-based drivers were drivers stemming from newly identified opportunities and emerging evidence. In recognition of national and international mental health sector reform initiatives, the peer workforce has grown internationally and across Australia [13]. Supporting and expanding the peer workforce is a key strategic direction of NSW’s Strategic Framework and Workforce Plan for Mental Health (2018–2022), aligning with similar priorities internationally [14].

In 2016, an Australian non-government organisation piloted a program called Hospital to Home. This small, local, peer worker-delivered program was designed to provide practical and emotional support to people as they transitioned from psychiatric in-patient units back to community living and to link people with community-based supports. An independent evaluation of this pilot identified that the program supported positive outcomes for participants in terms of recovery, wellbeing, and reduced re-hospitalisation [13].

Peer-STOC draws upon the priority to expand the peer workforce and upon the emerging evidence. Across NSW, 17 Peer-STOC programs each have one or two full-time equivalent peer worker positions. Peer-STOC peer workers are embedded within multi-disciplinary community or inpatient teams. Irrespective of where peer workers are based, Peer-STOC-delivered supports commence with hospital in-reach to build rapport, discuss wellbeing strategies, and collaboratively identify transitional supports needed. This transitional support is designed to be continued for approximately six weeks post-discharge. Supports provided are diverse, tailored to individual recovery-focused needs and wants. They include, for example, making community-based service and program referrals, helping people to attend and connect with communities and services of choice, social connection, someone to talk to, getting things organized, and getting out of the house. A primary aim of the Peer-STOC program was to reduce mental-health-related readmissions to hospital.

In the broader mixed-method study, we examined program impacts and outcomes as well as strengths and/or challenges to implementation, sustainability, and future expansion. Here, we report on service-user outcomes of the program from the perspectives of service-users themselves, Peer-STOC peer workers, and other workers from the mental health system that interacted with Peer-STOC.

## 2. Methods

This was an 18-month independent evaluation with data collected between November 2020 and July 2021. Our research team comprised research expertise both with (three) and without (three) lived experience of mental ill-health and mental health service use. A Lived Experience Advisory Panel (LEAP) supported development, interpretation, and translation aspects of the project. This paper reports on qualitative findings relating to service-user outcomes. A companion paper will report on quantitative findings. Ethics approval was obtained from Sydney Local Health District Human Research Ethics Committee (Protocol # 2020/ETH01054).

### 2.1. Recruitment

Flyer and email invitations to participate in the study were distributed by the 17 Peer-STOC managers. Current and previous Peer-STOC service-users, Peer-STOC peer workers, and other mental health workers and clinicians who interfaced with the Peer-STOC program were all invited to participate.

### 2.2. Data Collection

Data collection included both a brief open-ended online questionnaire and individual, in-depth semi-structured interviews. People could choose to engage in either or both. Both the questionnaire and interviews explored people’s experiences of Peer-STOC, its strengths, and limitations.

Interviews were conducted by two lived experience research team members and ranged from 30 min to 1.5 h. A flexible interview guide allowed participants to focus on aspects most pertinent to them, and researchers to ask follow-up questions [15]. Participants provided informed consent prior to the interview and were offered a small gift voucher as a thank you for their time and contribution. Interviews were conducted via phone or video-conferencing platform due to COVID restrictions and to enhance geographical reach. A distress protocol was developed for actions to be taken if a participant experienced psychological distress during interviews. However, this event did not arise and thus the protocol did not need to be actioned. Interviews were audio-recorded with participant consent. Recordings were transcribed verbatim and entered, along with questionnaire data into NVivo software for organisation and management during analyses.

### 2.3. Data Analysis

Data collection and analysis were conducted concurrently to allow interviewers to pursue lines of inquiry informed by earlier interviews and analyses. Data were thematically analysed inductively using constant comparative analysis [15]. Methodological rigour was enhanced through frequent reflective discussions between researchers and the LEAP team.

### 2.4. Participants

A total of 82 people completed the questionnaire: 12 service-users currently or previously engaged in Peer-STOC, 20 Peer-STOC peer workers and 50 other mental health workers such as clinicians who had engaged in some way with Peer-STOC. Interviews were conducted with 58 people: 17 service-users, 22 Peer-STOC peer-workers, and 19 other mental health workers. Questionnaires were anonymous; thus, questionnaire and interview participants could not be linked. However, some questionnaire participants also participated in interviews, thus the total number of individual participants is less than 140. Self-reported demographic details are provided in Table 1 below.

## 3. Results

Across interviews and questionnaires, service-users (SU), peer workers (PW), and other workers (OW) repeatedly and consistently described positive service-user outcomes or impacts. These included: (a) a better, less traumatic inpatient experience; (b) felt understood, cared about, and less alone; (c) easier to leave hospital; (d) easier to get back into life and daily routines; (e) built and re-established community connections; (f) gained new knowledge, strategies, and skills; and (g) felt more hopeful about my recovery. These themes are provided in Table 2 below with example quotes. To distinguish questionnaire from interview quotes, ‘q’ is used. Each theme is then detailed.

### 3.1. A Better, Less Traumatic Inpatient Experience

Repeatedly, service-users emphasised the positive impact of caring interactions and conversations with Peer-STOC peer workers on their overall in-patient experience. They described feeling comfortable, building a connection with, and trusting their peer worker. They talked about the peer worker empathy and shared understanding experienced at times when they were feeling distressed or hopeless on the ward: SU10 said: “*it was easy to talk to someone that had a lived experience… rather than doctors and medical staff*”. Similarly, SU6 said: “*having that experience, touching base with a peer worker… it made me feel at ease. It made me feel comfortable… they are really good assets for the hospital because… which gets back to this trust thing again …* [they are the] *go-betweens—between you and the doctor or you and any member of the hospital*”.

Other workers also repeatedly described positive impacts of Peer-STOC on service-users’ in-patient experience. They described the peer workers availability, individualised, caring interactions with service-users, and the value of their shared experience. Other workers said these, and the lack of a medical agenda during conversations, collectively reduced service-user distress. OW29, reflecting comments of others, explained: “*you’ve got clients that come into the ward that don’t want to be there… it can create an atmosphere that can be traumatic for people… a peer support worker is someone that can kind of remove them from the situation… and help them unpack what’s going on*”. OW28 said: “*the whole environment* [on the ward] *has got the potential for a lot of tension…* [the Peer-STOC worker]*, goes over there, and he even does things like make people cups of tea. So, it’s the least threatening or safest… It’s not to do with…‘You have to take these pills’, … His approach is, ‘What would you like me to do?’, or ‘What can I do to help you?’”*.

### 3.2. Felt Understood, Cared about and Less Alone

Service-users repeatedly said that because of their shared experience of mental ill-health and service use, their Peer-STOC workers made them feel understood, cared about and less alone. SU3 explained “*I have things in common with them*”. SU19 said “*my Peer-STOC worker was the one person I could really connect with*” and SU17 explained: “*he understands my situation…. He’s had his own experiences with mental health, so it’s just an* [opportunity] *to talk to someone with the ability to empathise with the situation*”.

Feeling understood and cared about led service-users to feel less alone. SU9 said: “*it helped me just to have a person that was interested in me, that I could talk to because I was very alone and isolated and fairly scared*”. SU3 said: “*It helps me … feel, like I’m not alone with my problem because sometimes…it feels like I’m the only one in the world or the universe with it*”. Some service-users emphasised how critically important connection with their peer worker was when they were alone and feeling hopeless or suicidal. Reflecting on service-user comments, PW21 said: “*you sit and talk to them for an hour, but that might have stopped them from picking up a blade and cutting themselves or, you know, taking too much medication… or whatever… you know, just that one conversation has made a difference to them*”.

### 3.3. Easier to Leave Hospital

Unsurprisingly, given this was a core program objective, almost all service-users talked about how Peer-STOC support made facing and managing discharge and the initial transition back into living at home easier. Several people described the fear they had about leaving inpatient unit supports when discharged. SU13 said she felt: “*like I was a little bird… that’s been looked after and then they let it out of the cage… you’ve been nurtured in the hospital environment, then* [they are] *releasing you back into the wild*”. Service-users said that having Peer-STOC involvement “[made] *get*[ting] *out of the hospital or going to the community very easy*” (SU1). SUq91 said that they: “*felt supported. It took the edge off the change, bringing a bit of the hospital into the outside world. Helped me with anxiety*”.

Some people compared positive experiences of Peer-STOC supported transition to previous experiences of discharge without follow-up support. SU17 said: “*in 2013… there was no sort of peer follow-up when I left* [hospital]*… I went straight from there to jail*”. SU10 said: “*I’ve had stints where I’ve* [been in hospital] *for three and a half months at a time and then had to go home to an empty house by myself and manage* [with] *no support. That’s been really scary… it has made a hell of a difference in the recovery and getting back to what’s normal… it’s made the journey and the process a lot easier*”. Peer-STOC peer workers and other workers, also repeatedly said the program provided service-users “*a softer landing and a softer transition* [back to community]” (OW13).

A number of service-users, said their transition home was eased by the continuity of a connection they had already established with the Peer-STOC peer worker while on the ward, SU1 explained: “*it’s good he* [peer worker] *went to the hospital because when I went out of the hospital, I think I was looking forward to seeing him again*”. SU10 said: “*It was just easier when you left hospital because you already had a rapport with that person… in hospital and you’ve* [already] *like spilled your guts* [so] *you don’t have to re-spill your guts*”. The value of continuity was also highlighted by other workers*:* “*they’re the only staff members who work across the in-patient and the community, so being able* to [say/know]… *‘I’m getting the support and engagement from somebody while I’m in the unit, and then I know that when I go home… I’m going to see that same person, and that familiar face… and they know me’*” (OW3).

### 3.4. Easier to Get Back into Life and Daily Routines

Service-users talked about the impact of Peer-STOC on their ability to get back into life after leaving hospital. They described their peer workers supporting them to get their home organised, to get out of the house and to start, or return, to previous, personally valued routines and activities. SU10 said: “*it really helped me to get back on my feet and in a routine once I got home… if I didn’t have that support, I think I probably wouldn’t have bounced back as quickly as I have*”. Similarly, SU4 said that after a few meetings with their peer worker: “*I sort of started to feel like I was looking after myself a bit better and… then I was ready to sort of start getting back to my old self and looking after myself again*”. Employment was part of re-establishing meaningful routine for several service-users, and they described ways that their peer worker facilitated this. SU1 for example said: “*when I left the hospital, he* [peer worker] *made an email to my boss… telling* [them] *that I want to go back to the company… and I was accepted again*”.

Other people said that having someone to encourage them, and to physically be with, made getting out of the house easier during the early days after being in hospital. SU9 said: “*then he started to meet me at the shopping centre…it got me out of the house. That’s what I needed… it gave me a person to be with*”. SU15 said: “*I had trouble leaving the house. He would take me to the community, and we’d have a coffee*”, and SU16 said: “*just having someone to come over and take me out… it’s better than going out somewhere alone*”.

Service-users described going back from hospital to homes that felt disorganised or overwhelming to manage. They described practical supports, suggestions, and connections that Peer-STOC peer workers provided to help them manage and become more organised. SU17 explained: “*I was very stressed and disorganised and… everything was sort of falling apart whereas now I feel pretty comfortable… He has helped me organise everything and take small steps but to accomplish big things*”. Peer-STOC peer workers also talked about supporting people to re-engage in routines as an important part of their role: “*a big part of* [what] *I see, like an integral part of that transition is helping people plan a routine when they’re in the community*” (PW8).

### 3.5. Built and Re-Established Community Connections

Every service-user we spoke to described having greater community connections because of their Peer-STOC engagement. Connections they described included: community mental health services and support; services and supports beyond the mental health system; and personal connections and relationships.

Creating new connections or re-establishing previous connections was not easy, and people repeatedly described the value of their peer worker going with them rather than just providing recommendations or referrals, because “*it helps with building confidence and getting there, because it’s an icebreaker*… *they know what’s going on*” (SU3). Other workers also highlighted the value of: “*the peer support worker who accompanies them, there’s … a bit more companionship about going to something that you might feel nervous about going to on your own*” (OW28).

Service-users, peer workers, and other workers all described Peer-STOC as bridging the gap between service-users and community mental health services: SU15 said their peer worker “*helped out with the relationship with community mental health… I really needed someone to really bridge the gap between where community health was coming from and where I was coming from*”. Similarly, OW16 said: “*It’s improved the engagement of clients with … their case manager, psychiatrist*”. OW29 explained that without the Peer-STOC peer worker, service-users “*might not engage with the case managers and staff or the hospital and it kind of becomes a revolving door*”.

Peer-STOC peer workers also supported service-users to establish better connections with a diverse range of other community-based services and resources outside of mental-health-specific services. These included disability supports, government agencies, public housing, and local charities to access life essentials such as accommodation, food, and clothing. SU17 said: “*he put me in touch with people like St Benedict’s where I can get a warm meal and hotels and places that are available… he did quite a bit of research for me to help me try and find places*”. At the time of the interview, SU17 said that through their Peer-STOC peer worker’s assistance, they were no longer homeless and staying in relatively stable accommodation. SU3 said that their peer worker: “*took me… just me and her, looking around* [charity shops]*… places where I can get cheap books… she took me to Salvation Army, and we inquired about when they* [were] *giving away food*”. Other workers also described these broader community connections. OW28, for example, saw Peer-STOC peer workers: “*help the client… look toward their recovery and their supports in the community that might not just be based around mental health services*”.

Peer workers also supported people to establish personally meaningful connections and to engage in personally meaningful activities beyond health or community services. These included educational activities, volunteering and employment, sport and recreation communities, programs, and activities. PW6: “*helped* [service-users] *enrol in educational programs… get involved in volunteer work*”. PW20 supported someone to connect with a community yoga group: “*I walked alongside with her,* [went] *to the first yoga class,* [to] *help her feel comfortable doing that … she found that really … beneficial*”. SU23 said: “*one thing I really, really miss is going to the beach coz I can’t walk on sand or anything anymore and then she told me… that she could take me down there to the surf club and we can find out about the information for the beach access wheelchair… to get from the sand to the water*”.

### 3.6. Gained New Knowledge, Strategies, and Skills

Service-users repeatedly described how they had, with Peer-STOC assistance, gained new knowledge, strategies, and skills. The new knowledge people repeatedly talked about, was awareness of community-based services and resources: “*he’d tell me about the resources available in the community. That was helpful*” (SU16). Some people described learning about online resources. SU3 said: “*she told me about … some good apps to use for mindfulness*”. SU24 felt: “*more knowledgeable on mental health and also more knowledgeable on getting a job*”.

Service-users also talked about gaining skills and strategies. SU13 explained that her Peer-STOC peer worker “*encouraged me to write a list … if you needed certain things done around the house or you needed to ask different people*”. SU14 said: “*she helped me a lot with lifestyle techniques*”. Peer-STOC peer workers also described supporting people to develop new strategies and skills, often by sharing their own, as well as encouraging service-users to recognise the skills and strategies they already had. PW5 described: “*reminding* [service-users] *that they have the tools, or building the tools with them, on how to create boundaries with other people and take charge of these conversations that they are worried about*”. PW9 had: “*connected with one consumer* [service-user] *who never had anyone to talk to about his voices at all… I was able to talk about strategies I have for managing voices that could hopefully help him*”. PWq62 valued: “*having the opportunity to… help* [people] *understand what they can do for themselves to help make their lives better for themselves*”. PW13 talked about: “*kind of helping them think more about themselves in terms of their strengths*”.

### 3.7. Felt More Hopeful about My Recovery

Service-users frequently explained that interactions with their Peer-STOC peer worker made them feel inspired and more hopeful about themselves and their own recovery, although ‘recovery’ was not a word they often used. Some examples of what people said include: “*She probably gave me hope when I was pretty down in the hospital*” (SU24). Another commented that engaging with their Peer-STOC peer work helped her see: “*there’s light at the end of the dark tunnel*” (SU3) and “*it did help me with my wellbeing and progress*” (SUq91). Peer-STOC peer workers’ recovery-oriented and strengths-based approach helped people reframe and feel more positive about themselves. SU21 said that working with their Peer-STOC peer worker: “[made] *me re-look at myself and things that I’ve done and achieved and how good they were… it made me stop and turn around and focus on the good in my life, not the bad*”.

Service-users also described feeling more self-confident because of their engagement with the Peer-STOC peer worker. SU6 said: “*they make me feel very, very reassured and they make me feel well. They make me feel confident and clear and you know in tune with my thoughts*”. SU9 said Peer-STOC: “*was of great assistance for me. It gave me some confidence that there was someone there. I think it was very good for my mental health*”. Similarly, SU17 said*:* “*my state of mind has improved tremendously*… *if I’m having any problems, it’s someone that I can talk to… together we can find solutions to problems*”.

Reflecting on service-user testimonies, Peer-STOC peer workers also noticed service-users they worked with became more hopeful, optimistic, and self-confident after contact with Peer-STOC: “*I see them walking away with more strength, more resilience, more positive about what they could achieve in their own lives*” (PW21). PW10 said: “*With that extra support we have been able to provide, you know,* [support] *people’s recovery a little and just help them to stay afloat and be a bit more resilient and hopefully prevent them from going back to hospital*”.

## 4. Discussion

These findings powerfully resonate with the connection and belonging, or relational elements of recovery [16,17,18]. Peer workers build connections with service-users through sharing common experiences, but also by demonstrating in various ways that they care about them. Many descriptions from service-users, peer workers, and other staff observing the service-user–peer worker relationship reflect the relational role of others in the recovery process, as highlighted by Topor and colleagues [19]: being available, doing more than expected, and doing something different than what was expected. Being available by phone, spending time for conversation, advocating, explaining, making cups of tea, going with people to appointments or activities rather than merely providing referrals or information are but a few of the examples of connection-building activities in which the Peer-STOC peer workers engaged.

Repeatedly peer workers and the Peer-STOC program were described as a ‘bridge’—a connector. Peer workers created bridges that enabled service-users to build connections within, across, and beyond mental health services as well as to natural communities of choice.

It was this connectedness, developed through the service-user’s relationship with their Peer-STOC peer worker that facilitated all seven outcomes: (a) a better, less traumatic inpatient experience; (b) felt understood, cared about, and less alone; (c) easier to leave hospital; (d) easier to back into life and daily routines; (e) built and re-established community connections; (f) gained new strategies, knowledge, understanding and skills; and (g) felt more hopeful about my recovery.

Beyond relational aspects, some authors emphasise the broader social or societal aspects of recovery [20]. These include having access to good material conditions such as secure and safe housing and financial security. The community connections fostered by peer workers in the Peer-STOC program enhanced people’s societal and economic recovery needs such as navigating and accessing housing departments and housing services, resolving neighbourhood conflicts, linking with food, furniture and clothing assistance services, and reconnecting with previous employers and employment.

## 5. Limitations

It is important to recognise the problems faced with service-user recruitment. Due to the ethics-driven requirement for no direct approach, service users were informed about the opportunity to participate in the study by program managers. The proportion of service users completing interviews and the questionnaire was disappointing. In addition, as with all qualitative research, it is important to recognise that the experiences and perspectives of those who participated might differ from those who did not. This is an Australian, NSW-based program. Further research exploring the experiences and outcomes of similar initiatives internationally would add further evidence of the value of peer-delivered programs supporting people transitioning from acute care back to community living.

## 6. Conclusions

Others have identified the valuable impacts and outcomes of peer-delivered programs [6]. These findings add to that body of evidence and emerging evidence of the particular value of recovery-focused peer-delivered supports provided to people before, during, and after they transition from inpatient psychiatric settings back to home [11,13]. These findings also evidence that other workers within the mental health system recognise the positive impact that this peer-delivered program has had on the recovery outcomes of service-users.

## Figures and Tables

**Table 1:** Demographic summary of participants.

*Stakeholder Group* Category		Interview	Questionnaire
*Service-users*		*n* = *17*	*n* = *12*
Gender	Female	9 (52.9%)	4 (33.3%)
	Male	7 (41.2%)	4 (33.3%)
	Non-binary/other identity	1 (5.9%)	0 (0.0%)
	Not stated	0 (0.0%)	4 (33.3%)
Age	18 to 29 years	2 (11.8%)	1 (8.3%)
	30 to 44 years	4 (23.5%)	4 (33.3%)
	45 to 64 years	10 (58.8%)	7 (58.3%)
	65 or over	1 (5.9%)	0 (0.0%)
Diagnosis (self-report)	Schizophrenia/Psychosis	-	4 (33.3%)
	Depression	-	6 (50.0%)
	Bipolar disorder	-	3 (25.0%)
	Anxiety	-	3 (25.0%)
	Borderline personality disorder	-	2 (16.7%)
	Other	-	2 (16.7%)
***Peer workers***		***n* = *22***	***n* = *20***
Gender	Female	11 (50.0%)	9 (45.0%)
	Male	7 (31.8%)	8 (40.0%)
	Non-binary/other identity	4 (18.2%)	3 (15.0%)
Age	18 to 29 years	3 (13.6%)	3 (15.0%)
	30 to 44 years	7 (31.8%)	7 (35.0%)
	45 to 64 years	12 (54.5%)	10 (50.0%)
Years as PW	Under 1 year	2 (9.1%)	3 (15.0%)
	Between 1 to 5 years	16 (72.7%)	12 (60.0%)
	More than 5 years	3 (13.6%)	5 (25.0%)
	Not reported	1 (4.5%)	
Years in Peer-STOC	Less than 1 year	5 (22.7%)	15 (75.0%)
	Greater than 1 year	16 (72.7%)	5 (25.0%)
	Not reported	1 (4.5%)	
***Other workers***		***n* = *19***	***n* = *50***
Gender	Female	16 (84.2%)	-
	Male	3 (15.8%)	-
Age	18 to 29 years	1 (5.3%)	-
	30 to 44 years	7 (36.8%)	-
	45 to 64 years	10 (52.6%)	-
	65 or over	1 (5.3%)	-
Background/training	Social Worker	1 (5.3%)	4 (8.0%)
	Occupational Therapist	1 (5.3%)	8 (16.0%)
	Nurse	6 (31.6%)	12 (24.0%)
	Psychologist	2 (10.5%)	8 (16.0%)
	Peer/Consumer worker	7 (36.8%)	9 (18.0%)
	Other	2 (10.5%)	10 (20.0%)
Worked with peer workers before PS	Yes	-	34 (68.0%)
	No	-	16 (32.0%)
Years working in MH	Less than 5 years	-	12 (24.0%)
	Between 5 and 10 years	-	11 (22.0%)
	More than 10 years	-	27 (54.0%)
Location	Inpatient	13 (68.4%)	15 (30.0%)
	Community	6 (31.6%)	31 (62.0%)
	Other	0 (0.0%)	4 (8.0%)

**Table 2:** Synthesis of service-user outcomes from all stakeholder perspectives.

SERVICE-USER OUTCOMES	
Themes	Example Quotes
A better, less traumatic inpatient experience	*“You’ve got clients that come into the ward that … don’t want to be there either so it can create an atmosphere that can be traumatic for people you know… Having like a peer support worker is like someone that can kind of remove them from the situation … and help them like unpack what’s going on”* (OW29) *“We did a lot of activities together… I was fully allowed to be sad or get angry or you know get a bit nostalgic in a safe environment where I wasn’t being judged on how much better I was getting… Loved my Peer-STOC worker”* (SU19)
Felt understood, cared about, and less alone	“[the Peer-STOC program] *gave me … someone to talk to that really understood where I was coming from”* (SU15) *“it’s just pure understanding and pure empathy”* (SU6) *“It helped me just to have a person that was interested in me that I could talk to because I was very alone and isolated and fairly scared”* (SU9)
Easier to leave hospital	*“I felt supported. It took the edge off the change, bringing a bit of the hospital into the outside world”* (SUq91) “[Made] *me get*[ting] *out of the hospital or going to the community very easy”* (SU1)
Easier to get back into life and daily routines	“*It really helped me to get back on my feet and in a routine once I got home… if I didn’t have that support, I think I probably wouldn’t have bounced back as quickly as I have”* (SU10) *“It’s just peace of mind. Like my head was a mess and it sort of helped me come back to reality and get more, start to get organised”* (SU17) *“He helped me go back to work easily”* (SUq31)
Built and re-established community connections	“[My Peer-STOC] *peer worker helped me in getting involved with the psychiatrist… also helped me for him to be in between me and the psychiatrist”* (SU1) *“We helped her connect with a psychologist”* (PW16) *“I’ve helped consumers enrol in educational programs… get involved in volunteer work, I’ve linked consumers in with clothing outlets… Even things like taking people to Oz Harvest”* (PW6)
Gained new knowledge, strategies, and skills	*“She helped me a lot with lifestyle techniques”* (SU14) *“he’d tell me about the resources available in the community. That was helpful”* (SU16) *“She told me about … some good apps to use for mindfulness”* (SU3) *“I think I’m more organised now… I’ve got these big plastic envelopes that we went and got at* [stationery shop] *and I put my bills and documents and medical documents in, and so … that actually helps, if I’m more organised I’m not as anxious”* (SU13)
Felt more hopeful about my recovery	*“She probably gave me hope when I was pretty down in the hospital”* (SU24) *“They make me feel very, very reassured and they make me feel well. They make me feel confident and clear and you know in tune with my thoughts”* (SU6) *“I see them* [consumers] *walking away with more strength, more resilience, more positive about what they could achieve in their own lives”* (PW21)

## Data Availability

The data presented in this study are available on request from the corresponding author.
